# Supplementary material for: Shared Treg dysregulation underlies psoriasis vulgaris and atopic dermatitis through the PHF19-PRC2 epigenetic axis
Source: Front Immunol. 2026 Aug 20;17:1932788. doi: 10.3389/fimmu.2026.1932788 (PMC13538097; doi:10.3389/fimmu.2026.1932788)
Supplement: Supplementary file 3 [file DataSheet3.zip › Additional files/Additional file 4/12.FAM118A.leaveoneout.pdf]

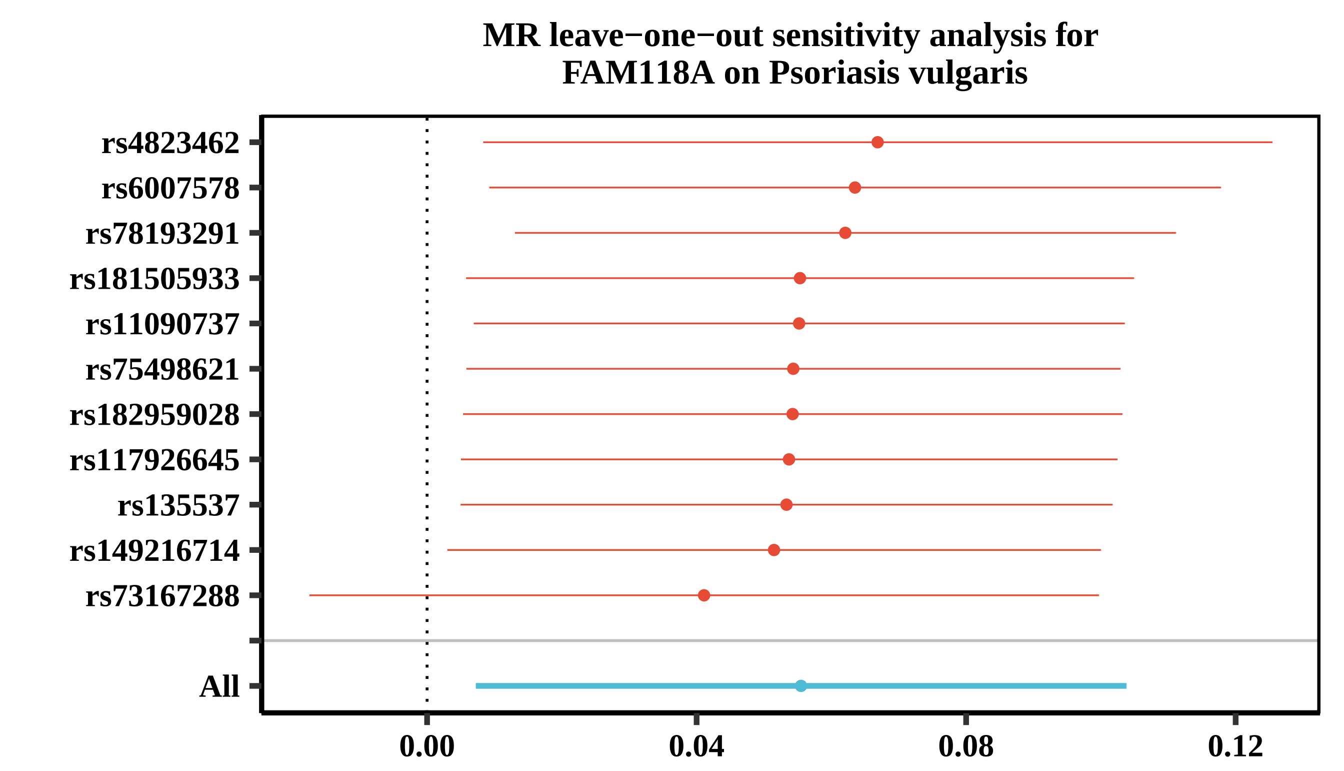

MR leave−one−out sensitivity analysis for
FAM118A on Psoriasis vulgaris
rs4823462
rs6007578
rs78193291
rs181505933
rs11090737
rs75498621
rs182959028
rs117926645
rs135537
rs149216714
rs73167288
All
0.00
0.04
0.08
0.12
